# Vehicle Detection Based on Probability Hypothesis Density Filter

**DOI:** 10.3390/s16040510

**Published:** 2016-04-09

**Authors:** Feihu Zhang, Alois Knoll

**Affiliations:** Robotics and Embedded Systems, Technische Universität München, 80333 München, Germany; knoll@in.tum.de

**Keywords:** LiDAR, vehicle detection

## Abstract

In the past decade, the developments of vehicle detection have been significantly improved. By utilizing cameras, vehicles can be detected in the Regions of Interest (ROI) in complex environments. However, vision techniques often suffer from false positives and limited field of view. In this paper, a LiDAR based vehicle detection approach is proposed by using the Probability Hypothesis Density (PHD) filter. The proposed approach consists of two phases: the hypothesis generation phase to detect potential objects and the hypothesis verification phase to classify objects. The performance of the proposed approach is evaluated in complex scenarios, compared with the state-of-the-art.

## 1. Introduction

Traffic accidents are a major cause of death worldwide. A study by the World Health Organization (WHO) reports that an estimated 1.2 million people die in traffic accidents every year, and up to 50 million people are injured [1]. Autonomous driving thus becomes significantly important in order to prevent accidents in traffic scenarios. However, it is still quite challenging for autonomous driving in all scenarios. For automotive manufacturers, the technology behind autonomous driving has been continually refined as a long-term goal, whereas the Advanced Driver Assistance System (ADAS) has been proposed as a short-term development to gradually improve road safety. Numerous ADAS functions have been developed to help drivers avoid accidents, improve driving efficiency, and reduce driver fatigue, in which vehicle detection plays an important role.

Most approaches rely on vision techniques to first detect Regions Of Interest (ROI) and then classify vehicles [2]. Khammari *et al.* use the Adaboost classification to detect vehicles [3]. Miller *et al.* and Paragios *et al.* have also utilized filtering techniques to detect vehicles [4,5]. Meanwhile, vehicle profile symmetry and the corresponding shadows are used in Reference [6]. However, vision techniques suffer from light intensities.

LiDAR is also widely used in vehicle detection. In contrast to vision sensors, LiDAR is robust against light intensities and offers a range of information [7,8,9,10,11]. Teichman *et al.* use the log odds estimators to recognize objects, where the performance is demonstrated in a large scale environment. Dominguez *et al.* demonstrate a data fusion platform for tracking vehicles [12]. Compared with vision sensors, LiDAR measurement often suffers from data association issues.

This paper extends our previous work to detect vehicles by using information from LiDAR, where objects are represented by the position and shape parameters (more details would be explained later). In Reference [13], we used the Difference of Normal (DoN) operator and the Random Hypersurface Model (RHM) to cluster the points cloud data and estimate the shape parameters [14,15]. To avoid the data association issue, the Probability Hypothesis Density (PHD) filter is proposed to detect vehicles based on Random Finite Set statistics (RFSs) [16]. In RFSs, several approaches are developed to avoid the data association issue, including the PHD filter, the Cardinalized PHD (CPHD) filter [17] and the Bernoulli filter [18]. The PHD filter propagates the probability hypothesis density function over the single target state space, whereas the CPHD filter also propagates the distribution of the target numbers (cardinality). By using the CPHD filter, the system requires more complex implementations and achieves more reliability in cardinality estimation. As the main goal of this paper is to estimate the states in a speed-critical environment, the PHD filter is thus considered. Unlike the PHD or CPHD filter, the multi-Bernoulli filter propagates the posterior target density. Although it has the same complexity as the PHD filter, the performance is better in highly nonlinear environments (it does not require the additional clustering step for state estimation) [18]. As the proposed RHM could be linearly implemented, the PHD filter is considered as the cheapest solution. The estimated states are then classified by the Support Vector Machine (SVM) to eliminate the non-vehicle objects.

The contributions are summarized as: first and foremost, the proposed solution achieves high performance in the presence of unknown data association environments. Furthermore, the shape parameter is first proposed to classify objects.

This paper is structured as follows: Section 2 describes the random hypersurface model, as well as the probability hypothesis density filter for hypothesis generation. Section 3 introduces the support vector machine for hypothesis verification. Section 4 demonstrates the performance of the experiments in urban environments. Finally, the paper is concluded in Section 5.

## 2. Hypothesis Generation

In the hypothesis generation phase, LiDAR measurements are filtered based on the Random Hypersurface Model (RHM). To further extend RHM in the presence of unknown data association scenarios, the Gaussian Mixture Probability Hypothesis Density (GMPHD) filter is proposed. Notice that objects are tracked and estimated in the 2D Cartesian coordinate system, whereas the depth information is unnecessarily required.

The result of the generation phase is then utilized for the verification phase to eliminate non-vehicles.

### 2.1. Random Hypersurface Model (RHM)

**Figure 1:**
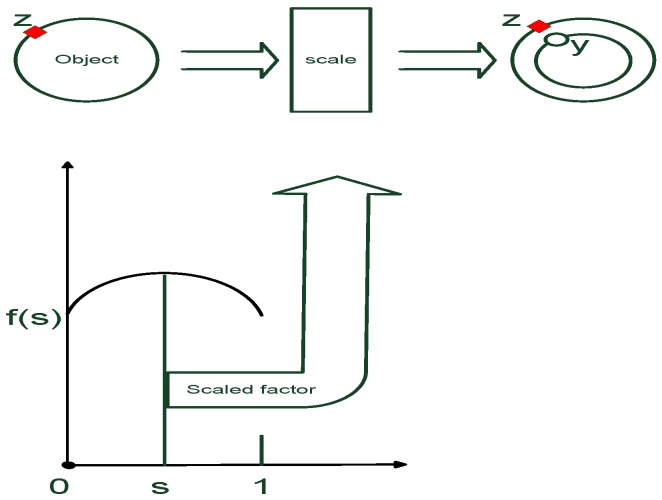
Random hypersurface model.

As illustrated in Figure 1, in a 2D Cartesian coordinate system, a point is considered as a scaled point with the factor within the range [0,1] drawn on the surface. Thus, the RHM is defined as:

$S({\bar{b}}_{k})$ denotes the surface which consists of both the shape parameter ${\bar{b}}_{k}$ and the center $c_{k}$. Each point that lies on the surface is represented as a scaled boundary when *s* is drawn from [0,1]:

(1) $$c_{k}+s\cdot \left(S\left({\bar{b}}_{k}\right)-c_{k}\right)$$

**Figure 2:**
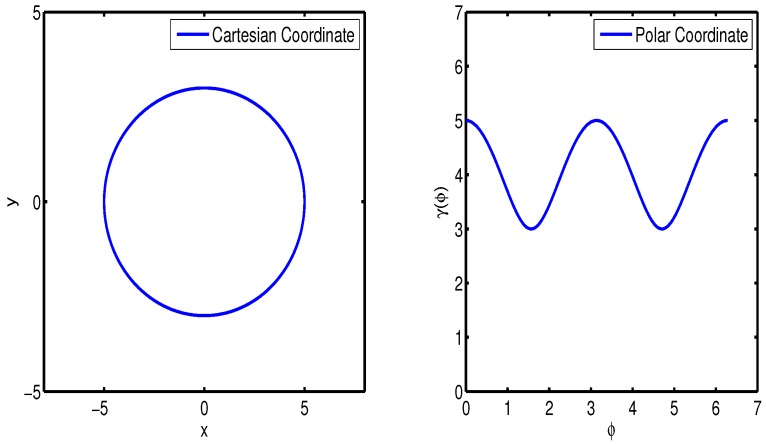
Example of star-convex object.

In Figure 2, r(ϕ) denotes the distance function calculated from the center to boundary on angle *φ* in the polar coordinate system.

Assuming r(ϕ) consists of the shape parameter ${\bar{b}}_{k}$ and the center $c_{k}$, the surface is represented as

(2) $$S\left({\bar{b}}_{k}\right)=\left\{s\cdot r\left({\bar{b}}_{k},\phi \right)\cdot e\left(\phi \right)+c_{k}|\phi \in \left[0,2\pi \right],s\in \left[0,1\right]\right\}$$

where $e\left(\phi \right):\;=\left[\begin{array}{c} \cos\phi \\ \sin\phi \end{array}\right]$ and $r({\bar{b}}_{k},\phi )$ denote the unit vector and the radial function in the form of the Fourier series, respectively. Due to its periodic proprieties, the Fourier series expansion of degree $N_{F}$ becomes

(3) $$r\left({\bar{b}}_{k},\phi \right)=a_{k}^{0}+\sum_{j=1}^{N_{F}}\left\{a_{k}^{j}\cos\left(j\phi \right)+b_{k}^{j}\sin\left(j\phi \right)\right\}$$

where ${\bar{b}}_{k}$ is given by

(4) $${\bar{b}}_{k}={\left[a_{k}^{0},a_{k}^{1},b_{k}^{1},\ldots ,a_{k}^{N_{F}},b_{k}^{N_{F}}\right]}^{T}$$

If *φ* is fixed, Equation (3) is represented as

(5) $$r\left({\bar{b}}_{k},\phi \right)=R\left(\phi \right)\cdot {\bar{b}}_{k}$$

where

(6) $$R\left(\phi \right)=[1,\cos\left(\phi \right),\sin\left(\phi \right),\ldots \;,\cos\left(N_{F}\phi \right),\sin\left(N_{F}\phi \right)]$$

Notice that a low number of Fourier coefficients encode rough information of the surface, whereas a larger number of coefficients give more details.

#### Bayes Filter

The RHM represents the shape information by using the Fourier coefficients, and the Bayes filter is utilized to calculate the corresponding parameters.

- Process model

Assuming the state $x_{k}$ denotes the Fourier descriptors ${\bar{b}}_{k}$ and does not drift against time, the process model is described as

(7) $$x_{k}=A_{k}x_{k-1}+w_{k}$$

where $A_{k}$ and $w_{k}$ denote the identity matrix and the process noise, respectively.

- Measurement model

As illustrated in Figure 1, a single measurement $y_{k}$ from LiDAR is originated from a surface boundary point $z_{k}$ with scaled factor s,

(8) $$y_{k}=f\left(z_{k},s\right)+v_{k},f\left(z_{k},s\right)\in S\left({\bar{b}}_{k}\right)$$

where $v_{k}$ denotes the measurement noise.

Using Equation (2), Equation (8) becomes

(9) $$\begin{array}{cc} y_{k}= & f\left(z_{k},s\right)+v_{k}\\ = & s\cdot r\left({\bar{b}}_{k},\phi \right)\cdot e\left(\phi \right)+c_{k}+v_{k}\\ := & h(x_{k},v_{k}) \end{array}$$

which maps the relationship between the state ${\bar{b}}_{k}$ and the measurement $y_{k}$.

Based on Equation (5), the measurement model is represented as

(10) $$y_{k}=s\cdot R\left(\phi \right)\cdot {\bar{b}}_{k}\cdot e\left(\phi \right)+c_{k}+v_{k}$$

with algebraic manipulations on Equation (10), we get

(11) $$\left|\right|y_{k}-c_{k}{\left|\right|}^{2}=s^{2}\cdot ||R\left(\phi \right)\cdot {\bar{b}}_{k}{\left|\right|}^{2}+2sR\left(\phi \right){\bar{b}}_{k}e{\left(\phi \right)}^{T}v_{k}+\left|\right|v_{k}{\left|\right|}^{2}$$

The measurement model is thus acquired as:

(12) $$\begin{array}{c} 0=s^{2}\cdot ||R\left(\phi \right)\cdot {\bar{b}}_{k}{\left|\right|}^{2}+2sR\left(\phi \right){\bar{b}}_{k}e{\left(\phi \right)}^{T}v_{k}+\left|\right|v_{k}{\left|\right|}^{2}-\left|\right|y_{k}-c_{k}{\left|\right|}^{2} \end{array}$$

where a pseudo measurement 0 is used to model the relationship between the state, the scaled factor, the measurement and its noise. The state $x_{k}$ convergences to the true Fourier descriptor ${\bar{b}}_{k}$ by updating with a large number of measurements.

Notice that the proposed measurement model is implemented in the 2D Cartesian coordinate system and is only effective on the backside of the target. In addition, the depth information from LiDAR measurement is unrequested.

**Figure 3:**
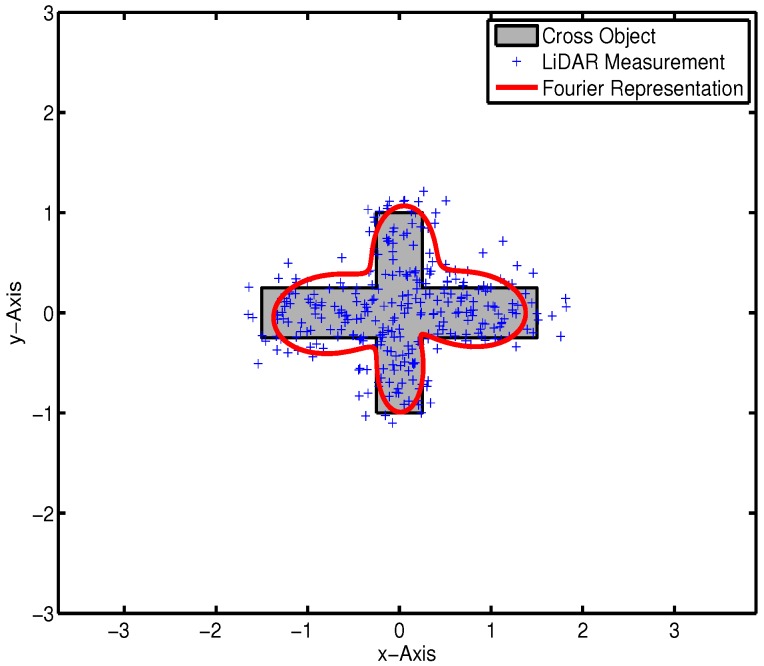
Bayesian estimation in a data association known environment.

Figure 3 exhibits the estimated result with a cross target, in which the state x and measurement *y* are represented by 13 Fourier coefficients and 2D Cartesian coordinates, respectively.

### 2.2. Probability Hypothesis Density (PHD) Filter

As illustrated in Figure 3, the RHM estimates the shape information by using the Bayes filter. The key challenge for practice implementation is data association. A traditional estimator operates in measurement-to-target known scenarios, where all assignments are confirmed. However, LiDAR provides a large number of measurements without any association information. In previous work, the Difference of Normal (DoN) operator is utilized as a preprocessing procedure to cluster measurements. The miss detection is quite high since the clustering process may also eliminate objects. Hence, the PHD filter is proposed to track objects in presence of unknown data association scenarios.

#### 2.2.1. Overview

**Figure 4:**
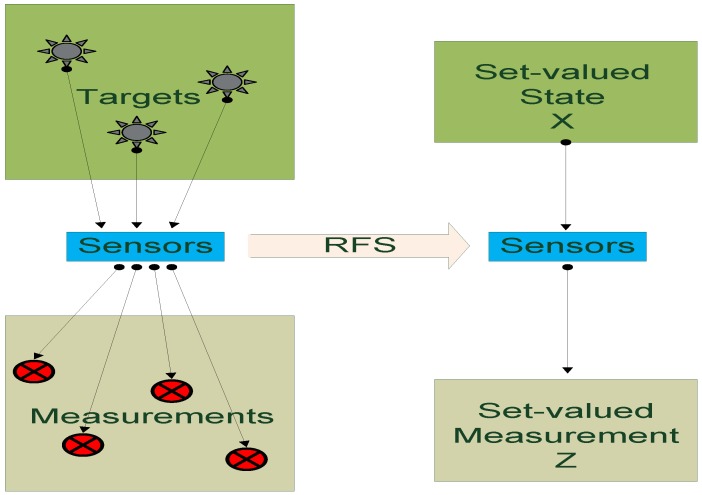
Set-valued states and set-valued observations.

The PHD filter is represented with the set-valued state and observation for multiple-object Bayesian filtering. All targets and observations are collected and represented in the set space, whereas the data association problem in traditional filtering domain is avoided. Figure 4 is a basic introduction of the RFS statistic. Compared to the single target filtering, the PHD filter relies on the random finite set statistics to process data in the set space level.

#### 2.2.2. Mathematic Background

Considering the survived targets $S_{k|k-1}$, the spontaneous targets $\sigma_{k}$ and the spawned targets $B_{k|k-1}$, the set-valued state is described as:

(13) $$X_{k}=\left[\underset{\zeta \in X_{k-1}}{⋃}S_{k|k-1}\left(\zeta \right)\right]\cup \left[\underset{\zeta \in X_{k-1}}{⋃}B_{k|k-1}\left(\zeta \right)\right]\cup \sigma_{k}$$

In addition, the set observation $Z_{k}$ consists of the reflections from both the targets $\theta_{k}\left(x\right)$ and the clutters $\kappa_{k}$.

(14) $$Z_{k}=\left[\underset{x\in X_{k}}{⋃}\theta_{k}\left(x\right)\right]\cup \kappa_{k}$$

Similar to Bayesian estimator, the PHD filter is also divided into prediction and update processes. Notice that *D* and $f_{k|k-1}\left(x|\zeta \right)$ denote the posterior density and the transition function, respectively. ζ is the previous state.

Thus, the prediction is represented as:

(15) $$D_{k|k-1}\left(x\right)=\left[\int \left[P_{S}\left(\zeta \right)f_{k|k-1}\left(x|\zeta \right)+\beta \left(x|\zeta \right)\right]D_{k-1}\left(\zeta \right)d\zeta \right]+\gamma_{k}$$

The intensity function is updated based on the measurement set $Z_{k}$:

(16) $$\begin{array}{c} D_{k}\left(x\right)=\left(1-P_{D}\right)D_{k|k-1}\left(x\right)+\sum_{z\in Z_{k}}\frac{P_{D}g_{k}\left(z_{i}|x\right)D_{k|k-1}\left(x\right)}{\kappa \left(z_{i}\right)+\int P_{D}g_{k}\left(z_{i}|\zeta \right)D_{k|k-1}\left(\zeta \right)d\zeta } \end{array}$$

where $g_{k}\left(z_{i}|x\right)$ and $P_{D}$ denote the likelihood function and the detection probability, respectively.

Notice that the predict function in Equation (15) are affected by targets, which enter the scene ($\gamma_{k}$), and survive from the previous time step $P_{S}$ and the spawn targets (β(x|ζ)).

The update function in Equation (16) corrects the prediction by using innovations from the observations. Notice that the clutter rate is also considered in the update function.

(17) $$N\left(k\right)=\int_{\Psi }D_{k|k}\left(x\right)dx$$

Equation (17) exhibits that the integration of the intensity function represents the number of targets. Meanwhile, the intensity is not a probability density and thus unnecessarily sums up to 1 [19].

The PHD recursions have multiple integrals with no closed form representation. Thus, the most common approach is to use Gaussian Mixture (GM)-PHD approximations [20]:

(18) $$f_{k|k-1}\left(x|\zeta \right)=N\left(x;F_{k-1}\zeta ,Q_{k-1}\right)$$

(19) $$g_{k}\left(z|x\right)=N\left(z;H_{k}x,R_{k}\right)$$

where z and x denote the current measurement and state, whereas ζ denotes the previous state. A Gaussian distribution is represented as N(·;m,P) with mean m and covariance *P*. $F_{k-1}$ and $Q_{k-1}$ denote the transition matrix and process covariance, respectively. $H_{k}$ and $R_{k}$ denote the observation matrix and observation noise covariance. Notice that the detection and survival probabilities are constant values:

(20) $$P_{S,k}\left(x\right)=P_{S},P_{D,k}\left(x\right)=P_{D}$$

Birth targets $\gamma_{k}$ are modeled as:

(21) $$\gamma_{k}\left(x\right)=\sum_{i=1}^{J_{\gamma ,k}}\omega_{\gamma ,k}^{\left(i\right)}N\left(x;m_{\gamma ,k}^{\left(i\right)},P_{\gamma ,k}^{\left(i\right)}\right)$$

where $\omega_{\gamma ,k}^{\left(i\right)}$, $P_{\gamma ,k}^{\left(i\right)}$, $m_{\gamma ,k}^{\left(i\right)}$ and $J_{\gamma ,k}$ denote the weight, covariance, mean and amount of the Gaussians.

Assuming the posterior intensity at time k−1 is a Gaussian mixture:

(22) $$D_{k-1}\left(x\right)=\sum_{i=1}^{J_{k-1}}\omega_{k-1}^{\left(i\right)}N\left(x;m_{k-1}^{\left(i\right)},P_{k-1}^{\left(i\right)}\right)$$

Equation (15) to time *k* is also a Gaussian mixture

$$D_{k|k-1}\left(x\right)=P_{S}\sum_{i=1}^{J_{k-1}}\omega_{k-1}^{\left(i\right)}N\left(x;m_{S,k|k-1}^{\left(i\right)},P_{S,k|k-1}^{\left(i\right)}\right)+\gamma_{k}\left(x\right)\;$$

$$\begin{array}{cc} & m_{S,k|k-1}^{\left(i\right)}=F_{k-1}m_{k-1}^{\left(i\right)},P_{S,k|k-1}^{\left(i\right)}=Q_{k-1}+F_{k-1}P_{k-1}^{\left(i\right)}F_{k-1}^{T} \end{array}$$

and the Equation (16) at time *k* is calculated as

(23) $$D_{k}\left(x\right)=\left(1-P_{D}\right)D_{k|k-1}\left(x\right)+\sum_{z\in Z_{k}}D_{D,k}\left(x;z\right)$$

where

$$D_{D,k}\left(x;z\right)=\sum_{j=1}^{J_{k|k-1}}\omega_{k}^{\left(j\right)}\left(z\right)N\left(x;m_{k|k}^{\left(j\right)}\left(z\right),P_{k|k}^{\left(j\right)}\right)$$

$$\omega_{k}^{j}\left(z\right)=\frac{P_{D}w_{k|k-1}^{\left(j\right)}q_{k}^{\left(j\right)}\left(z\right)}{\kappa_{k}\left(z\right)+P_{D}\sum_{l=1}^{J_{k|k-1}}w_{k|k-1}^{\left(l\right)}q_{k}^{\left(l\right)}\left(z\right)}$$

$$q_{k}^{\left(j\right)}\left(z\right)=N\left(z;H_{k}m_{k|k-1}^{\left(j\right)},H_{k}P_{k|k-1}^{\left(j\right)}H_{k}^{T}+R_{k}\right)$$

$$m_{k}^{\left(j\right)}\left(z\right)=m_{k|k-1}^{\left(j\right)}+K_{k}^{\left(j\right)}\left(z-H_{k}m_{k|k-1}^{\left(j\right)}\right)$$

$$P_{k}^{\left(j\right)}=\left[I-K_{k}^{\left(j\right)}H_{k}\right]P_{k|k-1}^{\left(j\right)}$$

$$K_{k}^{\left(j\right)}=P_{k|k-1}^{\left(j\right)}H_{k}^{T}{\left[H_{k}P_{k|k-1}^{\left(j\right)}H_{k}^{T}+R_{k}\right]}^{-1}$$

The GMPHD filter addresses the data association challenge in contrast to the standard Bayes filter. The process model Equation (18) and measurement model Equation (19) are equal to the RHM Bayes filter in Equations (7) and (8), whereas the implementation process is different.

### 2.3. RHM–GM–PHD Filter

In Section 2.2, the GMPHD filter is introduced for dealing with unknown data association issues. Notice that the standard GMPHD filter operates in condition that one reflection is received for each target per frame, called “Point Target (PT) tracking”. In the vehicle detection scenario, a large number of measurements would be collected from the surface of a single object, called “Extended Target (ET) tracking”. Therefore, the GMPHD filter should be redesigned for dealing with extended targets solely relying on LiDAR measurement.

The process model and measurement model are similar to the RHM Bayes filter in Equations (7) and (8). The prediction equation of the ET–GM–PHD filter are also the same as the standard GMPHD filter. The measurement update formulas for the ET–GM–PHD filter is introduced as:

(24) $$\begin{array}{c} D_{k|k}\left(x\right)=L_{Z_{k}}\left(x\right)D_{k|k-1}\left(x|Z\right) \end{array}$$

and the pseudo-likelihood function $L_{Z_{k}}\left(x\right)$ is defined as

(25) $$\begin{array}{c} L_{Z_{k}}\left(x\right)=1-\left(1-e^{-\gamma (x)}\right)P_{D}\left(x\right)+e^{-\gamma (x)}P_{D}\left(x\right)\sum_{p∠Z_{k}}w_{p}\sum_{W\in p}\frac{\gamma {\left(x\right)}^{\left|W\right|}}{d_{W}}\prod_{z\in W}\frac{\phi_{z}\left(x\right)}{\lambda_{k}c_{k}\left(z\right)} \end{array}$$

where $\lambda_{k}c_{k}\left(z\right)$ is the mean number of clutter measurements, $c_{k}\left(z\right)$ is the spatial distribution of the clutter, notation $p∠Z_{k}$ denotes that *p* partitions the measurement set $Z_{k}$ into non-empty cells *W*, notation W∈p denotes that *W* is a cell in the partition *p*, $w_{p}$ and $d_{W}$ denote the non-negative coefficients for each partition and cell, and ϕ(x) denotes the same likelihood function for a single measurement in Equation (12).

Here,

(26) $$\begin{array}{c} w_{p}=\frac{\prod_{W\in p}d_{W}}{\sum_{p^{{}^{\prime }}∠Z_{k}}\prod_{W^{{}^{\prime }}\in p^{{}^{\prime }}}d_{W^{{}^{\prime }}}} \end{array}$$

and

(27) $$\begin{array}{c} d_{W}=\delta_{|W|,1}+D_{k|k-1}\left[e^{-\gamma }\gamma^{\left|W\right|}P_{D}\prod_{z\in W}\frac{\phi_{z}}{\lambda_{k}c_{k}\left(z\right)}\right] \end{array}$$

where $\delta_{i,j}$ is the Kronecker delta function and |W| is the number of measurements in cell *W*. More details of the implementation process could be found in [21,22].

Hence, the ET–GM–PHD filter tracks the potential objects by solely relying on LiDAR without any association or cluster process. The estimated states represent the potential objects and would be filtered again by the support vector machine to eliminate non-vehicle objects.

## 3. Hypothesis Verification

To eliminate the outliers, the support vector machine is utilized to classify the vehicle and non-vehicle Fourier coefficients.

### 3.1. Support Vector Machine (SVM)

**Figure 5:**
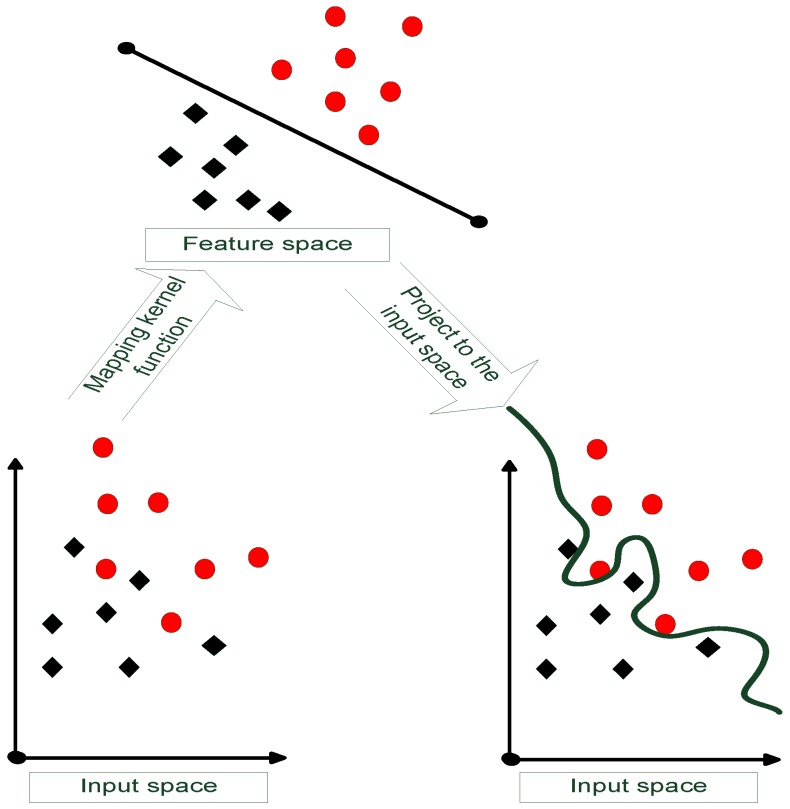
The concept of support vector machine (SVM)

As exhibited in Figure 5, the SVM is proposed to obtain classifiers with good generalization [23]. The mathematical background is introduced as follows:

For $x_{i}\in R^{n}$ with respect to the classification $y_{i}\in \left\{-1,+1\right\},i=1,\cdots ,k$, the hyperplane is defined as:

(28) $$f\left(x\right)=w^{T}x+b$$

to linearly separate each data. Notice that x, *w* and *b* denote the input vector, weight vector and the bias, respectively. Hence a maximum margin is found to separate positive class from negative class based on

(29) $$f\left(x\right)=\mathrm{sgn}\left(w^{T}x+b\right)$$

The calculation of the hyperplane is subjected to the following constraints:

$$\mathrm{Minimize}\;\frac{1}{2}{\left||w|\right|}^{2}$$

$$\mathrm{Subject}\;\mathrm{to}\;y_{i}\left(w_{i}x_{i}+b\right)\geq 0$$

and the classification performance relies on the optimization as:

$$\mathrm{Maximize}\;\sum_{i=1}^{k}\alpha_{i}-\frac{1}{2}\sum_{i,j=0}^{k}\alpha_{i}\alpha_{j}y_{i}y_{j}K\left(x_{i},x_{j}\right)$$

$$\mathrm{Subject}\;\mathrm{to}\;\sum_{i=1}^{k}y_{i}\alpha_{i}=0,\;\alpha_{i}\geq 0\;for\;i=1,\ldots ,k$$

where $K(x_{i},x_{j})$ and $\alpha_{i}$ denote the Kernel function and Lagrange multiplier, respectively. Notice that if the data can not be separated linearly, the kernel function changes according to

(30) $$K\left(x_{i},x_{j}\right)=K\left(x_{i},x_{j}\right)+\frac{1}{C}\delta_{ij}$$

where *C* denotes the penalize parameter.

### 3.2. Implementation Detail

During the implementation, there are still issues in both the generation and verification phases.

- ET–GM–PHD Implementation

To track objects in multiple frames, the alignment issue should also be considered. Thus the state to be tracked in the ET–GM–PHD filter is $x_{k}=[v_{x},v_{y},x,y,({\bar{b}}_{k}{)}^{T}{]}^{T}$, where ${\left[v_{x},v_{y},x,y,\right]}^{T}$ is the normal state of the object centroid in the 2D Cartesian coordinate system and $\left({\bar{b}}_{k}\right)$ is the shape parameter by using 13 Fourier descriptors. Although a higher number of Fourier descriptors returns more details of the surface, the computation performance and robustness is unsatisfied. In this paper, 13 Fourier descriptors are selected based on the experience during the experiment.

Each object follows the liner Gaussian dynamic Equation (18) with the following configurations:

(31) $$F_{k}=\text{diag}\left(A_{k},I_{13}\right),\;A_{k}=\left[\begin{array}{cc} I_{2} & 0_{2}\\ I_{2} & I_{2} \end{array}\right]$$

(32) $$Q_{k}=\text{diag}\left(C_{k},0.03I_{13}\right),\;C_{k}=\left[\begin{array}{cc} I_{2} & \frac{0_{2}}{2}\\ \frac{0_{2}}{2} & \frac{1}{4} \end{array}\right]$$

where $I_{n}$ and $0_{n}$ denote the n×n identity and zero matrix, respectively. The measurement covariance is given using parameters diag[0.5,…,0.5].

Regarding the measurement model, it follows the non-linear Gaussian dynamic Equation (19) and is described as:

$$\begin{array}{c} 0=s^{2}\cdot ||R\left(\phi \right)\cdot {\bar{b}}_{k}{\left|\right|}^{2}+2sR\left(\phi \right){\bar{b}}_{k}e{\left(\phi \right)}^{T}v_{k}+\left|\right|v_{k}{\left|\right|}^{2}-\left|\right|y_{k}-m_{k}{\left|\right|}^{2} \end{array}$$

where *s* is a Gaussian function with mean 0.5 and variance 0.03. The birth intensity of the PHD filter is given according to the geometry information. To reduce the computation complexity, measurements which fall into the road regions are utilized to initialize the vehicles. The birth intensity is thus given by

(33) $$\gamma_{k}\left(x\right)=0.1N\left(x;m_{\gamma ,k}^{1},P_{\gamma ,k}^{1}\right)+0.1N\left(x;m_{\gamma ,k}^{2},P_{\gamma ,k}^{2}\right)+0.1N\left(x;m_{\gamma ,k}^{3},P_{\gamma ,k}^{3}\right)$$

where $m_{\gamma ,k}^{1}=\left[0,0,0,1,2,0,\ldots ,0\right]$, $m_{\gamma ,k}^{2}=\left[0,0,-5,1,2,0,\ldots ,0\right]$ and $m_{\gamma ,k}^{3}=\left[0,0,5,1,2,0,\ldots ,0\right]$ represents the vehicles in the middle (0,1), left (−5,1) and right (5,1), respectively. $P_{\gamma ,k}^{1}=P_{\gamma ,k}^{2}=P_{\gamma ,k}^{3}=\mathrm{diag}\left[2,2,2,2,0.3,\ldots ,0.3\right]$.

- SVM Implementation

The KITTI dataset is utilized for training the classifier, which provides a set of 5000 training frames with 1893 manually labeled objects (car, van, tram, misc, pedestrian, cyclist, trunk and so on). For each object, the original measurements (all objects are labeled with a 2D box, where measurement-to-target association is confirmed) are projected to the 2D Cartesian coordinate system around its original point. The 13 Fourier coefficients are then calculated by using the standard nonlinear Kalman filter [15]. Instead of multiple categories, objects are only considered as vehicles and non-vehicles (actually it is divided by cars and non-cars). Then, the SVM is trained by collecting Fourier coefficients from all calculated objects. In further evaluation, another 2000 frames of test data is utilized to guarantee the performance both quantitatively and qualitatively. The SVM is implemented in both the training and test phases without cross-validation.

- Key Parameters and Open Issues

During the ET–GM–PHD process, potential objects are collected in the set-valued state. The Fourier coefficients describe the shape information, and the position vector represents the location. The estimated objects are also shown by using bounding boxes, where the mean point uses the position and the width/height are calculated based on the Fourier coefficients (the Fourier coefficients represent the rough shapes in polar coordinate systems, in which the width and height of the boundary box are calculated by setting the *φ* equals to 0 and $\frac{\pi }{2}$). Since the PHD filter addresses the data association issue, the points cloud data is directly utilized to estimate the set-valued states. No further cluster process is required. Meanwhile, for each single measurement, the probabilities of both detection and survival are constant and no more than 1. In addition, the PHD filter may estimate close objects as single objects mainly due to the scale factor *s*. For the RHM model, measurements are considered as random draws from boundaries with different scaling factors in the range [0,1]. When objects are close to each other, it is quite challenging to distinguish them.

During the SVM process, the calculated coefficients from objects are utilized to eliminate the outliers. Since most cars have a similar width/length rate, the car-labeled objects are treated as vehicles and the rest are non-vehicles. Furthermore, the 13 Fourier coefficients have been found to be quite challenging for linearly separating vehicles and non-vehicles. To better train the SVM, the radial based kernel function is utilized as:

(34) $$K\left(x_{i},x_{j}\right)=\exp\left(\frac{-\left|\right|x_{i}-x_{j}{\left|\right|}^{2}}{2\sigma^{2}}\right)$$

where *σ* affects distributing complexity in the feature space.

## 4. Experiment Evaluation

To evaluate the approach quantitatively and qualitatively, the KIT dataset is utilized and compared with the state-of-the-art [24]. During the experiment, the proposed approach is implemented in Matlab with 2 Cores@3 GHz, and the average time is 5 s per frame.

**Figure 6:**
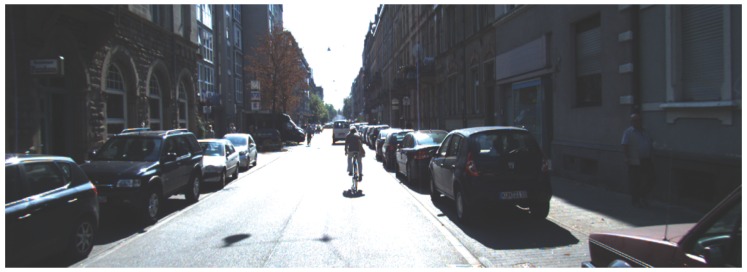
Original data from image view.

**Figure 7:**
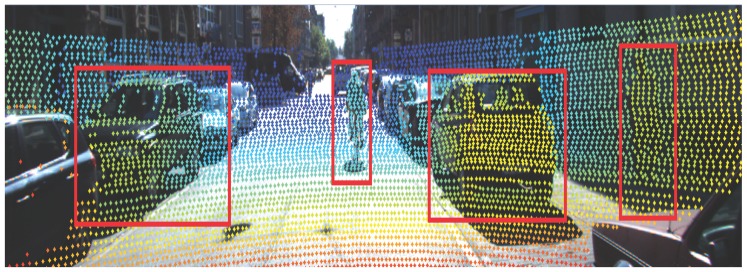
Result on hypothesis generation phase.

**Figure 8:**
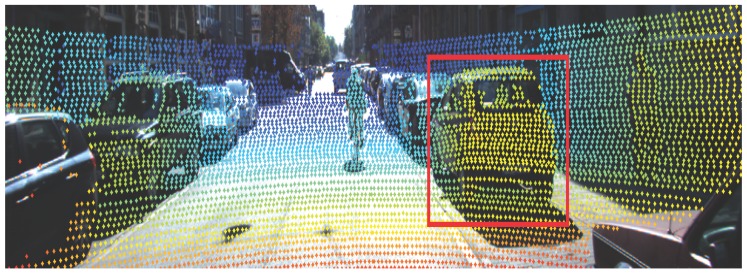
Result on hypothesis verification phase.

Figure 6, Figure 7 and Figure 8 demonstrate the detection performance based on the proposed approach in one scenario. In Figure 6, a bicycle is fully observed in the middle of the road. On the left side, a parking car is observed with partial occlusion. On the right side, both car and pedestrian are fully observed. As illustrated in Figure 7, potential objects are detected based on the RHM–ET–PHD filter. It is observed that the proposed approach extracts the potential objects based on the geometry of the road, where the birth model plays an important role. The extracted objects are drawn by boundary boxes calculated by the set-valued state (the center point is based on the position, and the width/height are based on the Fourier coefficients). Afterwards, the SVM is utilized to eliminate the non-vehicle objects. Figure 8 shows the results of the verification phase. Due to the occluded issues, it is observed that the left vehicle is also eliminated.

Table 1 demonstrates the overall performance, in contrast to the state-of-the-art, in all scenarios from the KITTI dataset. Although there are also approaches using cameras, the evaluation focuses on the algorithms which only use LiDAR measurements.

As illustrated in Table 1, moderate, easy and hard denote the occlusion level of vehicles, with respect to partly occluded, fully visible and difficult to see. Among the references, the proposed approach achieves a high performance for the easy category and poor performance for both moderate and hard categories. In easy scenarios, all vehicles are fully observed and the corresponding reflections on surfaces are uniformly distributed. Hence, the proposed approach can track and estimate the states successfully, and the final classification process has high performance. In moderate and hard scenarios, the corresponding performance drops significantly in both the generation and verification processes. For PHD filter, although it detects the potential objects, the calculated Fourier coefficients are strongly influenced by the invisible measurements. For SVM classification, the training process mainly relies on the visible measurements to calculate Fourier coefficients.

Nevertheless, compared with the overall performance in easy scenarios, the proposed approach still improves almost 20%–40%.

As a summary, the contributions are concluded as follows: first and foremost, the proposed framework solely relies on LiDAR measurements for vehicle detection in the presence of unknown data association environments. Furthermore, the Fourier coefficient is first proposed for object classification and concluded with high performance for fully visible vehicles.

## 5. Conclusions

Vehicle detection is important for developing driver assistance systems. To address the data association problem that suffers from points cloud data, the Probability Hypothesis Density (PHD) filter is proposed in this paper. The proposed scheme utilizes contour information for classification. The evaluation results illustrate a high performance in contrast to the state-of-the-art techniques.

Future work focuses on the improvement of detecting occluded vehicles.

## Figures and Tables

**Table 1:** Performance of the vehicle detection approach compared with the state-of-the-art.

Method	Ours	Vote3D [25]	CSoR [26]	mBoW [27]
Easy	75%	57%	35%	36%
Moderate	15%	48%	26%	24%
Hard	3%	43%	23%	18%
Average time	5 s	0.5 s	3.5 s	10 s
